# From Presumed Leiomyoma to Stump: Laparoendoscopic Single-Site Hysterectomy with Contained Morcellation for a Large Unsuspected Borderline Smooth Muscle Tumor

**DOI:** 10.3390/diagnostics16121760

**Published:** 2026-06-07

**Authors:** Kai-Hsiang Chang, Yen-Chang Chen, Dah-Ching Ding

**Affiliations:** 1 Department of Obstetrics and Gynecology, Hualien Tzu Chi Hospital, Buddhist Tzu Chi Medical Foundation, Tzu Chi University, Hualien 970, Taiwan; 102311106@gms.tcu.edu.tw; 2 Division of Digital Pathology, Department of Anatomical Pathology, Hualien Tzu Chi Hospital, Buddhist Tzu Chi Medical Foundation, Tzu Chi University, Hualien 970, Taiwan; s92312129@gmail.com; 3 Department of Pathology, School of Medicine, Tzu Chi University, Hualien 970, Taiwan; 4 Institute of Medical Sciences, Tzu Chi University, Hualien 970, Taiwan

**Keywords:** leiomyoma, smooth muscle tumor of uncertain malignant potential, menorrhagia, nulliparity, young

## Abstract

**Background:** Smooth muscle tumors of uncertain malignant potential (STUMPs) are rare uterine neoplasms occupying the diagnostic continuum between benign leiomyoma and overtly malignant leiomyosarcoma. Their preoperative identification remains beyond the capability of current imaging modalities, and the diagnosis is almost invariably established through postoperative histopathological examination. The natural history of STUMP is highly variable, with recurrence rates ranging from approximately 7–27% and a documented potential for malignant transformation, underscoring the need for accurate pathological classification and long-term surveillance. **Case Presentation:** A 40-year-old woman with a history of hypertension presented with a 5-month history of progressive lower abdominal distension, urinary frequency, and menorrhagia. Transabdominal ultrasonography identified a large uterine fundal mass measuring approximately 10.89 × 8.96 cm. Preoperative laboratory findings demonstrated microcytic anemia. She underwent laparo-endoscopic single-site supracervical hysterectomy with bilateral salpingectomy. Histopathological examination revealed spindle cells with bland to mildly atypical nuclei, a mitotic count of <10/10 high-power fields, and focal necrosis, consistent with a diagnosis of STUMP. The patient remained free of recurrence over a 2-year follow-up period. **Conclusions:** This case illustrates the diagnostic challenge posed by STUMP in the preoperative setting and highlights the critical importance of thorough histopathological evaluation of all uterine smooth muscle tumor specimens. Minimally invasive hysterectomy with in-bag morcellation represents a feasible surgical approach, and long-term oncological surveillance is warranted given the risk of late recurrence and malignant transformation. Clinicians should maintain a heightened index of suspicion for borderline smooth muscle tumors when evaluating large or symptomatic uterine masses in premenopausal women.

## 1. Introduction

A uterine smooth muscle tumor of uncertain malignant potential (STUMP) is a rare “gray-zone” entity characterized by unpredictable postoperative behavior and the absence of standardized surveillance strategies [1]. It is a subtype of uterine smooth muscle neoplasms defined by pathologic findings as intermediate between benign leiomyoma and malignant leiomyosarcoma (LMS); clinically, its presentation is comparable to that of leiomyoma, and the diagnosis is typically established postoperatively [2]. These tumors are morphologically heterogeneous and diagnostically challenging, with recurrence rates ranging from 7% to 28% and a higher propensity for recurrence observed in epithelioid and myxoid subtypes, and recurrent tumors may demonstrate histologic similarity to the initial STUMP or may fulfill criteria for LMS [3].

STUMP is currently diagnosed on the basis of three histologic parameters: mitotic index, cytologic atypia, and coagulative tumor cell necrosis, and the World Health Organization (WHO) classification defines these tumors as neither clearly benign nor malignant, reflecting a spectrum of clinical behavior [4]. According to Bell et al.’s Stanford criteria, LMS requires at least two of the following: diffuse moderate-to-severe atypia, a mitotic count of ≥10 mitotic figures per 10 high-power fields (HPFs), and tumor cell necrosis. Tumors that exhibit an unusual combination of these features without satisfying LMS thresholds are appropriately classified as STUMP. The variability in histologic appearance, immunohistochemical profile, and clinical outcome of STUMP makes its diagnosis particularly difficult, and the differing criteria adopted by pathologists further compromise diagnostic accuracy [5]. Immunohistochemical markers including p53, p16, Ki-67, and progesterone receptor (PR) have been investigated as adjuncts to morphologic assessment; among these, no single marker has proved robust enough to reliably separate STUMP from leiomyoma variants, although combined use of p16 and Bcl-2 has been proposed to help distinguish equivocal cases larger than 5 cm with at least moderate atypia and hyaline necrosis [6].

Total hysterectomy with or without bilateral salpingo-oophorectomy remains the standard of care for patients who have completed childbearing; myomectomy may be considered for younger patients with preserved fertility goals, although several studies have documented recurrence and distant metastases even following apparently complete resection [7]. A large, multicenter Italian cohort of 401 women demonstrated that definitive surgery was associated with significantly longer recurrence-free survival than fertility-sparing procedures, although overall survival remained excellent and comparable across surgical strategies with nearly all patients alive at last follow-up [8]. Among histopathological and surgical risk factors for recurrence, morcellation has been associated with shorter recurrence-free survival and should be avoided when STUMP is suspected preoperatively; epithelioid features, high proliferation activity, low PR expression, and diffuse p16 staining also confer increased recurrence risk [9]. For patients pursuing fertility-sparing surgery, a multicenter study of 106 women reported that approximately 22% experienced tumor recurrence, though most cases were non-cancerous [10]. Among those who actively attempted conception, 57.4% achieved pregnancy, supporting fertility-preserving myomectomy as a reasonable option under close long-term surveillance [10].

We report a case of STUMP occurring in a nulliparous woman presenting with progressive abdominal mass enlargement and lower abdominal discomfort. The primary novelty of this case lies in demonstrating the safe and feasible application of minimally invasive hysteretomy with contained in-bag morcellation in a patient with a large, unsuspected STUMP. This case highlights the inherent preoperative diagnostic challenge posed by STUMP, the oncological rationale for using tissue containment systems during morcellation, and the feasibility of minimally invasive approaches for bulky uterine specimens in the absence of preoperative suspicion of malignancy.

## 2. Case

### 2.1. Ethics

The patient provided written informed consent for the publication of this case report, including all clinical data and images.

### 2.2. Chief Complaint

An enlarging uterine mass with lower abdominal distension was noted over a 5-month period.

### 2.3. Present Illness

A 40-year-old woman presented with a progressively enlarging uterine tumor associated with lower abdominal discomfort. Her last menstrual period was 18 March 2025. She had a known history of hypertension managed with antihypertensive medication and no prior surgical history. Symptoms had been present since November 2023, prompting outpatient evaluation and subsequent admission for surgical planning.

On physical examination, vital signs were stable and the level of consciousness was normal. Abdominal examination revealed lower abdominal tenderness without fever, gastrointestinal symptoms (nausea, vomiting, diarrhea), or history of trauma or external injury. The patient reported a progressive increase in mass size accompanied by urinary frequency and heavier menstrual flow over the preceding year. Given the symptomatic burden and tumor growth, she elected to proceed with surgical intervention.

Transabdominal sonography demonstrated an anteverted uterus measuring 9 × 6 cm with a large fundal myoma measuring 10.8 × 12 cm. Endometrial thickness was 7.7 mm. Bilateral adnexa were not visualized, and no free fluid was identified in the cul-de-sac.

Based on the clinical and sonographic findings, she was admitted under the impression of an enlarged uterine myoma with abdominal pain. Laparo-endoscopic single-site supracervical hysterectomy with bilateral salpingectomy (LESS LSH + BS) was planned.

### 2.4. Medical History

The patient had a history of hypertension, managed with amlodipine (Norvasc, Pfizer Australia Pty Limited, West Ryde, NSW, Australia).

### 2.5. Laboratory Data

Preoperative blood work revealed microcytic anemia: white blood cell count 7360/μL, hemoglobin 12.1 g/dL, mean corpuscular volume 67.4 fL, and platelet count 269,000/μL.

### 2.6. Image Study

**Figure 1:**
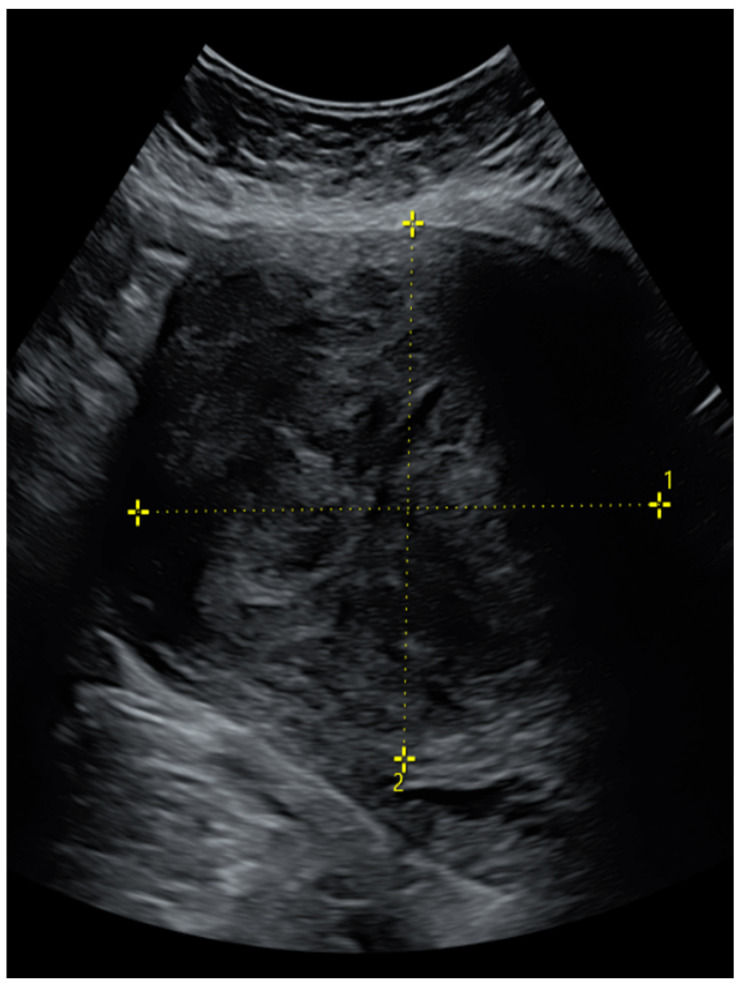
Uterine tumor by trans-abdominal sonography, size approximately 10.89 × 8.96 cm (Longitudinal view, 1: width, 2: height).

Transabdominal ultrasonography demonstrated a uterine tumor measuring approximately 10.89 × 8.96 cm (Figure 1). Preoperative grayscale ultrasound revealed a large, heterogeneous uterine mass with mixed echogenicity, comprising both low- and high-echogenic areas, without evidence of calcification (Figure 1). Color Doppler assessment was not performed during the imaging evaluation.

### 2.7. Operation and Hospital Course

Prophylactic antibiotics (Cefazolin 1 g) were administered intravenously 30 min prior to surgery. The patient underwent LESS LSH + BS. The surgical specimen was placed into a tissue containment system (Unimax Endo Pocket, Unimax Medical Systems Inc., Xindian, New Taipei City, Taiwan, ROC), and in-bag morcellation using a cold knife was performed. Total operation time was 3 h and 14 min, with an estimated blood loss of 50 mL. The in-bag deployment time was 13 min and 57 s, and morcellation time was 1 h and 30 min. The morcellated uterine specimen weighed 1105 g. The primary technical challenge was the prolonged morcellation time (1 h and 30 min) necessitated by the large specimen size; however, no intraoperative complications, bag rupture, or conversion to laparotomy occurred.

**Figure 2:**
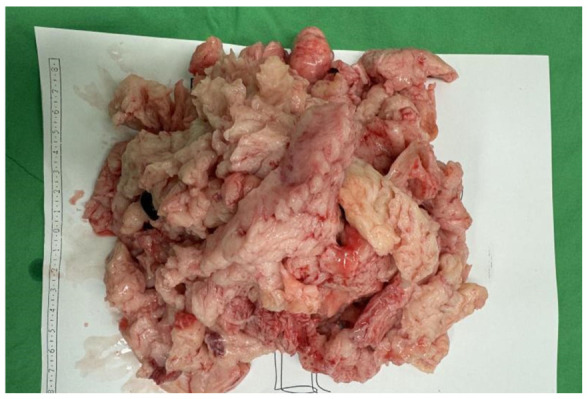
Gross picture of the tumor.

Gross examination revealed multiple irregular tissue fragments with a firm to rubbery consistency (Figure 2). The cut surfaces displayed a predominantly white to tan-yellow appearance with a whorled pattern, characteristic of smooth muscle origin. Focal areas of pale-yellow discoloration were noted, possibly representing early degenerative change. No macroscopic foci of hemorrhagic necrosis or coagulative tumor cell necrosis were identified on gross examination. No grossly visible capsular disruption was noted.

The postoperative course was unremarkable. Pain was well controlled with a numeric rating scale score of 2 at postoperative 24 h, decreasing to 1 thereafter. The patient resumed oral intake and ambulation without difficulty. No febrile episodes, wound complications, or other adverse events were noted. She was discharged on postoperative day 3 in stable condition.

### 2.8. Pathology

**Figure 3:**
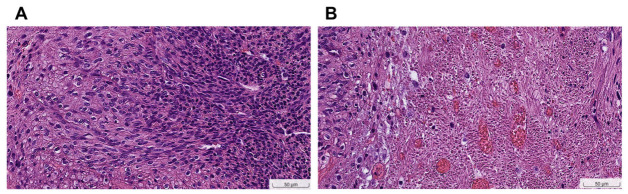
Histology of STUMP. (**A**) Spindle cells with bland to mildly atypical nuclei; (**B**) Focal necrosis. Scale bar = 50 μm.

Histopathological examination revealed a smooth muscle tumor composed of spindle cells with none to mildly atypical nuclei. Mitotic activity was low, averaging 0–1 mitosis/mm^2^ (fewer than 4 mitoses/mm^2^). Focal tumor cell necrosis was identified in the absence of moderate to severe atypia or elevated mitotic activity. In accordance with the WHO Classification of Tumours of Female Genital Organs, these findings fulfill the criteria for WHO STUMP Scenario 1—a smooth muscle tumor with tumor cell necrosis but without other worrisome histological features—consistent with a diagnosis of smooth muscle tumor of uncertain malignant potential (STUMP) (Figure 3).

### 2.9. Diagnosis

The patient was diagnosed with STUMP.

### 2.10. Follow-Up

The patient was followed up in the outpatient clinic. No evidence of recurrence or significant findings was observed over the 2-year follow-up period.

## 3. Discussion

### 3.1. Brief Summary of Novelty

The principal value of this case report is its demonstration that minimally invasive surgery with contained morcellation can be performed safely in the setting of a large, unsuspected STUMP. As STUMP cannot be distinguished from leiomyoma on preoperative imaging or clinical assessment alone, surgeons must be prepared for this diagnosis at the time of pathological review. The use of a tissue containment system in this case ensured oncological safety despite the unexpected borderline nature of the tumor, and supports the broader adoption of contained morcellation as a standard precaution in minimally invasive uterine surgery.

### 3.2. STUMPs

Uterine smooth muscle tumors represent a heterogeneous spectrum of mesenchymal neoplasms, ranging from the entirely benign leiomyoma to the overtly malignant LMS [1]. Situated between these two ends of the biological continuum lies a diagnostically challenging entity known as STUMP, a diagnostically challenging entity reflecting incomplete understanding of its natural history and the inherent limitations of histopathological classification [2]. This reflects the typical clinical scenario of STUMP diagnosed postoperatively after presumed leiomyoma surgery [3]. This discussion reviews the current literature pertaining to the definition, diagnostic criteria, clinicopathological features, surgical management, and surveillance strategies relevant to uterine STUMP.

### 3.3. Preoperative Diagnostic Challenges

The preoperative diagnosis of STUMP remains elusive with currently available imaging modalities [4]. Conventional ultrasonography, the most widely used first-line tool for evaluating uterine masses, cannot reliably differentiate STUMP from leiomyoma based on morphological features alone [2]. In the present case, transabdominal sonography demonstrated a large, heterogeneous uterine mass with mixed echogenicity and no calcifications, findings indistinguishable from those of a degenerating leiomyoma. MRI offers superior soft tissue contrast and may provide additional morphological detail; however, STUMPs do not consistently exhibit an MRI phenotype distinct from that of cellular or degenerated leiomyomas, and significant overlap exists between STUMP and other uterine smooth muscle tumors on both conventional and advanced quantitative MRI sequences [5]. In this case, MRI was not performed, as ultrasound provided sufficient information for surgical planning and MRI is not routinely indicated under our institutional protocol or reimbursed by Taiwan’s National Health Insurance program in the absence of specific clinical criteria. Taken together, the imaging findings in this case underscore the fundamental limitation that no currently available modality can reliably identify STUMP preoperatively, and that histopathological examination remains the diagnostic gold standard.

Serum biomarkers, including lactate dehydrogenase (LDH) and its isoforms, neutrophil-to-lymphocyte ratio, and platelet-to-lymphocyte ratio, have been explored as adjuncts for distinguishing leiomyosarcoma from leiomyoma; however, their diagnostic role in STUMP specifically has not been established [6]. Composite clinical scoring systems incorporating these biomarkers alongside clinical features such as tumor diameter, abnormal uterine bleeding, and rapid growth have shown promising discriminatory performance for leiomyosarcoma [6], but none has been validated for STUMP detection. Similarly, fluorodeoxyglucose positron emission tomography lacks sufficient sensitivity and specificity for routine preoperative evaluation of uterine smooth muscle tumors [7]. In the present case, no preoperative biomarker panel or advanced imaging was performed, as the clinical presentation was consistent with a large leiomyoma and no features prompted suspicion of a borderline or malignant tumor. This underscores the fundamental diagnostic challenge of STUMP: in the absence of reliable preoperative tools, the diagnosis invariably depends on histopathological examination of the surgical specimen, reinforcing the importance of thorough pathological assessment in all cases of hysterectomy or myomectomy performed for uterine smooth muscle tumors [8].

### 3.4. Pathological Classification and Histological Features

The histopathological diagnosis of STUMP requires systematic evaluation of three principal parameters: cytological atypia, mitotic activity, and tumor cell necrosis [2].

The classification of necrosis type represents the central diagnostic challenge in this case. The tumor’s large size and rapid clinical growth over five months suggest that ischemic (infarct-type) necrosis resulting from outgrowth of vascular supply is the most clinically plausible mechanism [1]. However, distinguishing histologically between infarct-type necrosis and coagulative tumor cell necrosis (CTCN) in borderline specimens is notoriously difficult, even among experienced gynecologic pathologists. Infarct-type necrosis is characterized by a gradual transition zone with surrounding hyalinization, whereas CTCN demonstrates an abrupt transition, ghost cell outlines, and nuclear debris—features associated with leiomyosarcoma [9]. When this distinction cannot be made with certainty, the diagnosis of STUMP is appropriate, reflecting the inherent biological and histological uncertainty of these tumors. It is this diagnostic ambiguity, rather than a definitive identification of either necrosis type, that underscores the importance of strict long-term follow-up in STUMP patients.

The present case exhibited focal necrosis with bland to mildly atypical nuclei and a mitotic index <10/10 HPF (averaging 0–1 mitosis/mm^2^). This pattern represents a common and particularly challenging STUMP subtype in which tumors with focal necrosis cannot be definitively characterized as coagulative tumor cell necrosis or hyaline necrosis. In such cases, expert pathological review and, where available, consultation with gynecological pathology specialists is strongly recommended. Immunohistochemical markers, including p16, p53, Ki-67, and h-caldesmon, could be investigated as potential adjuncts to morphological assessment in the classification of smooth muscle tumors [10].

### 3.5. Surgical Management

Since STUMP is almost invariably diagnosed postoperatively, its surgical management is largely determined by the clinical context of the initial operation [8]. Following hysterectomy with negative resection margins and no extrauterine disease, as in the present case, surgical management is considered complete [11]. For premenopausal patients diagnosed with STUMP after myomectomy who desire uterine preservation, management becomes considerably more complex; completion hysterectomy is advocated by several authors, particularly when necrosis cannot be reliably classified or atypia is moderate to severe, though individualized multidisciplinary decision-making remains the preferred approach given the absence of prospective data [11].

A critical surgical consideration is the risk of intraperitoneal tumor cell dissemination during morcellation of an unsuspected STUMP or leiomyosarcoma [12]. Following the FDA’s 2014 black box warning against power morcellation in women with known or suspected uterine malignancy [12], contained morcellation systems have been developed as a mitigation strategy, though their long-term oncological safety has not been definitively established [13,14]. In the present case, LESS LSH + BS was performed using cold knife in-bag morcellation within a closed tissue containment system, thereby avoiding peritoneal spillage despite the unanticipated STUMP diagnosis. This case demonstrates that minimally invasive surgery with contained morcellation is technically feasible and oncologically prudent for large uterine specimens when preoperative malignancy is not suspected, and supports its broader adoption as a precautionary standard in minimally invasive uterine surgery.

### 3.6. Prognosis and Recurrence

The natural history of STUMP is characterized by considerable biological variability. While the majority of patients remain disease-free following definitive surgical treatment, recurrence rates ranging from approximately 7% to 27% have been reported, with recurrent lesions occasionally exhibiting malignant transformation to leiomyosarcoma [15]. Histological features associated with increased recurrence risk include coagulative tumor cell necrosis, moderate to severe cytological atypia, elevated mitotic counts, epithelioid or myxoid subtypes, and diffuse p16 positivity, while surgical morcellation without containment has been independently associated with shorter recurrence-free survival [16]. In the present case, the large tumor size and histologically ambiguous necrosis type represent features warranting heightened vigilance, although the absence of extrauterine disease, negative resection margins, and use of contained morcellation are reassuring prognostic indicators. The patient demonstrated no evidence of recurrence over a two-year follow-up period. However, continued long-term surveillance remains essential given the documented risk of late recurrence and malignant transformation in this tumor category.

### 3.7. Surveillance and Follow-Up Recommendations

There is currently no consensus-based guideline for postoperative surveillance of STUMP, owing to the rarity of the diagnosis and the paucity of prospective data [17]. Most recommendations are extrapolated from surveillance protocols for low-grade uterine sarcomas and expert opinion [18]. Clinical follow-up at regular intervals, including physical examination and symptom assessment, is universally recommended. Patients should be counseled about the symptoms of recurrence, including new pelvic pain, abdominal distension, and respiratory symptoms suggestive of pulmonary metastasis, and encouraged to seek prompt medical evaluation for new or concerning symptoms [19].

### 3.8. Previous Reported Case Summary

The studies included in Table 1 were selected through a narrative literature search of PubMed using the terms “uterine STUMP,” “smooth muscle tumor of uncertain malignant potential,” and “uterine smooth muscle tumor borderline,” without a formal systematic review protocol. Inclusion was based on the following criteria: (1) studies reporting original clinicopathological data on uterine STUMP, including patient demographics, surgical approach, histopathological findings, and oncological outcomes; (2) availability of sufficient detail to permit meaningful comparison with the present case; and (3) representation of the breadth of the available literature, spanning large multicenter retrospective cohorts, single-center series, and individual case reports.

A summary of key published studies on uterine STUMP, including their clinicopathological features and oncological outcomes, is presented in Table 1. Published case series on uterine STUMP reveal consistent clinicopathological patterns. STUMP predominantly affects premenopausal women, with a mean age at diagnosis in the early-to-mid-forties, and presenting symptoms are virtually indistinguishable from those of benign leiomyoma [3]. The diagnosis is almost invariably established postoperatively on histopathological examination, as illustrated by the present case. Recurrence rates range from approximately 14% to 22% across series, with a subset exhibiting malignant transformation to leiomyosarcoma, underscoring the unpredictable biological behavior inherent to this diagnostic category [20]. Adverse prognostic factors identified across multiple cohorts include epithelioid histological subtype, elevated Ki-67, low PR expression, diffuse p16 positivity, and surgical morcellation without containment—the last of which was specifically avoided in the present case through the use of a closed in-bag system [13]. Given this risk profile, long-term structured surveillance—typically clinical and radiological assessment every six months for five years and annually thereafter—is warranted for all patients regardless of surgical approach, and multidisciplinary individualized management remains the cornerstone of care [21].

**Table 1:** Summary of key studies on uterine smooth muscle tumors of uncertain malignant potential (STUMP).

Study (Year)	Study Type	*N*	Mean Age (Years)	Chief Complaint/Presentation	Surgery	Histopathology	Recurrence/Outcome	Key Finding/Conclusion
Atay et al. (2026) J Obstet Gynaecol Res [3]	Retrospective single-center	27	43 (28–54)	Symptomatic uterine masses (menorrhagia, pelvic pressure); mostly premenopausal	Hysterectomy or myomectomy; minimally invasive and open approaches	STUMP per Stanford/WHO criteria; varied atypia, mitotic count, necrosis patterns	Late and occasional malignant recurrences documented; overall favorable if hysterectomy	STUMP shows heterogeneous postoperative behavior; long-term surveillance essential; uterus-sparing feasible in selected patients
Leone Roberti Maggiore et al. (2025) Hum Reprod Open [20]	Multicenter retrospective (13 Italian centers)	106	35.3 ± 6.8 (fertility-sparing cohort)	Symptomatic uterine mass; all desired fertility preservation	Fertility-sparing surgery (myomectomy open 69.8%, laparoscopy 20.8%, hysteroscopy 9.4%)	STUMP [22]; histology of recurrence: leiomyoma/STUMP 91.3%, LMS 8.7%	21.7% recurrence; median time to recurrence 18 months; 1 death (LMS recurrence); pregnancy rate 57.4% among those who tried	Fertility-sparing surgery feasible; pregnancy after STUMP associated with higher recurrence risk (63% vs. 7.6%)
Borella et al. (2022) Ann Surg Oncol [16]	Multicenter retrospective (5 tertiary centers, Italy/France)	87 (large series 2000–2020)	46 ± 10	Incidental/symptomatic uterine smooth muscle tumors	Hysterectomy or myomectomy; open and laparoscopic	STUMP; adverse features: epithelioid subtype, high Ki-67, low PR, diffuse p16	Morcellation, epithelioid features, high proliferative activity, low PR, diffuse p16 = increased recurrence; shorter RFS	Morcellation should be avoided; epithelioid features and IHC markers predict recurrence; individualized surveillance needed
Richtarova et al. (2023) Int J Gynecol Cancer [17]	Retrospective single-center	46	Median 36 (range 18–48)	Symptomatic uterine mass; all desired fertility	Myomectomy (fertility-sparing); reoperation (hysterectomy or re-myomectomy) for recurrence	STUMP (coagulative necrosis, low mitotic activity, without significant atypia in most)	Median follow-up 47.6 months; recurrence rate low; 20/46 achieved pregnancy; 19 live births	Myomectomy oncologically safe short-term; patients may not require immediate hysterectomy; close follow-up mandatory
Şahin et al. (2019) J Gynecol Oncol [21]	Dual-institution retrospective	57	Median 42 (range 16–75)	Symptomatic uterine mass (menorrhagia, pelvic pain, pressure)	Hysterectomy or myomectomy	STUMP per Stanford criteria; 1 case recurred as LMS after 14 months	14% recurrence (8/57); 7 recurred as STUMP, 1 as LMS; median follow-up 57 months	Fertility-sparing approaches feasible; recurrence may occur; LMS transformation possible
Basaran et al. (2018) Int J Gynecol Cancer [23]	Multicenter retrospective (centralized slide review)	21 (of 46 reviewed)	Mean 43 (range 20–64)	Symptomatic uterine masses; preoperative diagnosis leiomyoma in most	Hysterectomy or uterus-conserving surgery	STUMP confirmed on centralized review; significant reclassification rate (original diagnoses varied)	19% recurrence (4/21); 3 recurred as LMS; 1 death; mean follow-up 65.9 months	Centralized expert pathology review critical; recurrences may be aggressive with multiple relapses and death
Yordanov et al. (2020) Prz Menopauzalny [24]	Single-center retrospective + literature review	14 (single center series)	Median 45.4	Heavy menstrual bleeding most common; rapid growth uncommon	Hysterectomy (recommended); morcellation avoided	STUMP; ultrasound non-diagnostic; MRI limited specificity	5-year OS 92–100%; recurrence risk low but present; no lethal outcomes in their series	STUMP almost always unexpected postoperative diagnosis; morcellation associated with worse outcomes; follow-up q6 months ×5 years then annually
Lapresa-Alcalde et al. (2023) Diseases [19]	Retrospective case series (single hospital)	4	Mean 48 (range 36–67)	Abnormal uterine bleeding, compressive symptoms, pelvic mass (incl. residual cervix after prior subtotal hysterectomy)	Laparotomy; total hysterectomy (*n* = 2), subtotal hysterectomy (*n* = 1), cervical excision (*n* = 1)	STUMP [22] focal necrosis and mild atypia in most cases	No recurrence in follow-up period; all patients alive	STUMP can arise from residual uterine tissue; laparotomic approach used in all; long-term surveillance essential
Hughes et al. (2018) Int J Womens Health [5]	Case report	1	20	Menorrhagia, large fibroid uterus; nulliparous; fertility preservation desired	Myomectomy (Pfannenstiel); prior Goserelin (GnRH agonist) with volume reduction	STUMP (subserosal fibroid); Stanford criteria; mild atypia with focal necrosis	No recurrence at follow-up; patient preserved fertility	STUMP can occur in very young women; GnRH agonist pre-treatment effective; fertility-sparing myomectomy appropriate in carefully selected cases
Present case (2026) Diagnostics	Case report	1	40	Menorrhagia, urinary frequency, progressive lower abdominal distension (5 months); hypertension; nulliparous; virgin	LESS LSH + BS; no morcellation	STUMP: spindle cells, mild atypia, focal necrosis, mitotic index <10/10 HPF	No recurrence at 2-year follow-up	Large symptomatic STUMP managed safely by minimally invasive LESS approach without morcellation; unexpected histological diagnosis reinforces need for thorough pathological evaluation

Abbreviations: GnRH, gonadotropin-releasing hormone agonist; HPF, high-power fields; IHC, immunohistochemistry; LESS LSH + BS, laparo-endoscopic single-site supracervical hysterectomy with bilateral salpingectomy; LMS, leiomyosarcoma; MRI, magnetic resonance imaging; OS, overall survival; PR, progesterone receptor; RFS, recurrence-free survival; STUMP, smooth muscle tumor of uncertain malignant potential; WHO, World Health Organization. Studies were selected through a narrative literature search and include representative publications across a range of study designs and sample sizes. Inclusion criteria required reporting of original clinicopathological data on uterine STUMP with sufficient detail for comparison, including surgical approach, histopathological findings, and oncological outcomes.

### 3.9. Limitation

Color Doppler vascularity assessment was not performed, precluding characterization of internal blood flow patterns. Nonetheless, as no imaging criterion has demonstrated sufficient specificity to differentiate STUMP from other uterine smooth muscle tumors, this omission is unlikely to have affected clinical decision-making. MRI was not performed in this case due to a combination of clinical, institutional, and healthcare system factors: ultrasound provided adequate surgical planning information, MRI for uterine mass evaluation is not routinely reimbursed under Taiwan’s National Health Insurance program in the absence of specific indications, and our institutional preoperative protocol is ultrasound-based. While MRI may offer additional morphological detail, its inability to reliably distinguish STUMP from other uterine smooth muscle tumors on imaging alone means this omission is unlikely to have affected the diagnostic or clinical outcome.

In the present case, IHC staining could not be performed due to the unavailability of the archived tissue block. While we recognize the adjunctive value of p16, p53, Ki-67, ER, PR, and PHH3 in ambiguous smooth muscle tumors, the STUMP diagnosis was established on H&E morphology alone by an experienced gynecological pathologist, in accordance with established criteria. This case highlights key real-world diagnostic challenges. Accurate mitotic counting is particularly difficult in hypercellular or suboptimally fixed specimens; in such settings, PHH3 immunohistochemistry—cited as “helpful to evaluate mitoses” in uncertain cases by the WHO Classification of Tumors (5th ed., 2020) [22]—should be considered when the mitotic index approaches diagnostic thresholds. Additionally, STUMP overlaps morphologically with leiomyoma variants and leiomyosarcoma, requiring careful integration of cellularity, atypia, mitotic count, and coagulative necrosis. Adequate fixation, generous tumor sampling, and gynecological pathology specialist consultation are strongly recommended in diagnostically ambiguous cases.

### 3.10. Contribution to the Literature and Future Directions

This case adds to the literature by demonstrating the feasibility and oncological safety of LESS with cold knife in-bag contained morcellation for a large, unsuspected STUMP—an approach not widely reported in this specific context. It further reinforces that STUMP cannot be anticipated preoperatively regardless of tumor size, underscoring the importance of routine histopathological examination of all hysterectomy and myomectomy specimens. Future studies should prioritize prospective multicenter registries with standardized WHO-based histopathological reporting, long-term outcome data on contained morcellation systems in unsuspected borderline and malignant smooth muscle tumors, and consensus guidelines addressing surveillance protocols, indications for completion hysterectomy, and management of recurrent STUMP with malignant transformation.

## 4. Conclusions

STUMP represents a diagnostically and clinically challenging entity within the spectrum of uterine smooth muscle tumors. Its preoperative identification remains largely beyond the capability of current imaging technology, and the diagnosis is almost invariably established by postoperative histopathological examination. Accurate pathological classification, with particular attention to the characterization of necrosis type, degree of atypia, and mitotic activity, is essential to guide subsequent management decisions. Surgical treatment by hysterectomy is considered definitive for most patients, and minimally invasive approaches are appropriate when performed with in-bag morcellation. Long-term oncological surveillance is warranted given the documented, albeit relatively low risk of recurrence and malignant transformation. The present case contributes to the growing body of literature on STUMP and highlights the importance of maintaining a heightened index of suspicion for borderline or malignant smooth muscle tumors when evaluating large or symptomatic uterine masses in premenopausal women.

## Data Availability

All data are presented in the article.

## Abbreviations

The following abbreviations are used in this manuscript:

AUC	area under the curve
CT	computed tomography
HPF	high-power field
LDH	lactate dehydrogenase
LESS LSH + BS	laparo-endoscopic single-site supracervical hysterectomy with bilateral salpingectomy
LMS	leiomyosarcoma
MRI	magnetic resonance imaging
pLMS	pre-operative LMS
PR	progesterone receptor
STUMP	smooth muscle tumor of uncertain malignant potential
WHO	World Health Organization
